# Supplementary figures and images for: A secreted LysM effector protects fungal hyphae through chitin-dependent homodimer polymerization
Source: PLoS Pathog. 2020 Jun 23;16(6):e1008652. doi: 10.1371/journal.ppat.1008652 (PMC7337405; doi:10.1371/journal.ppat.1008652)

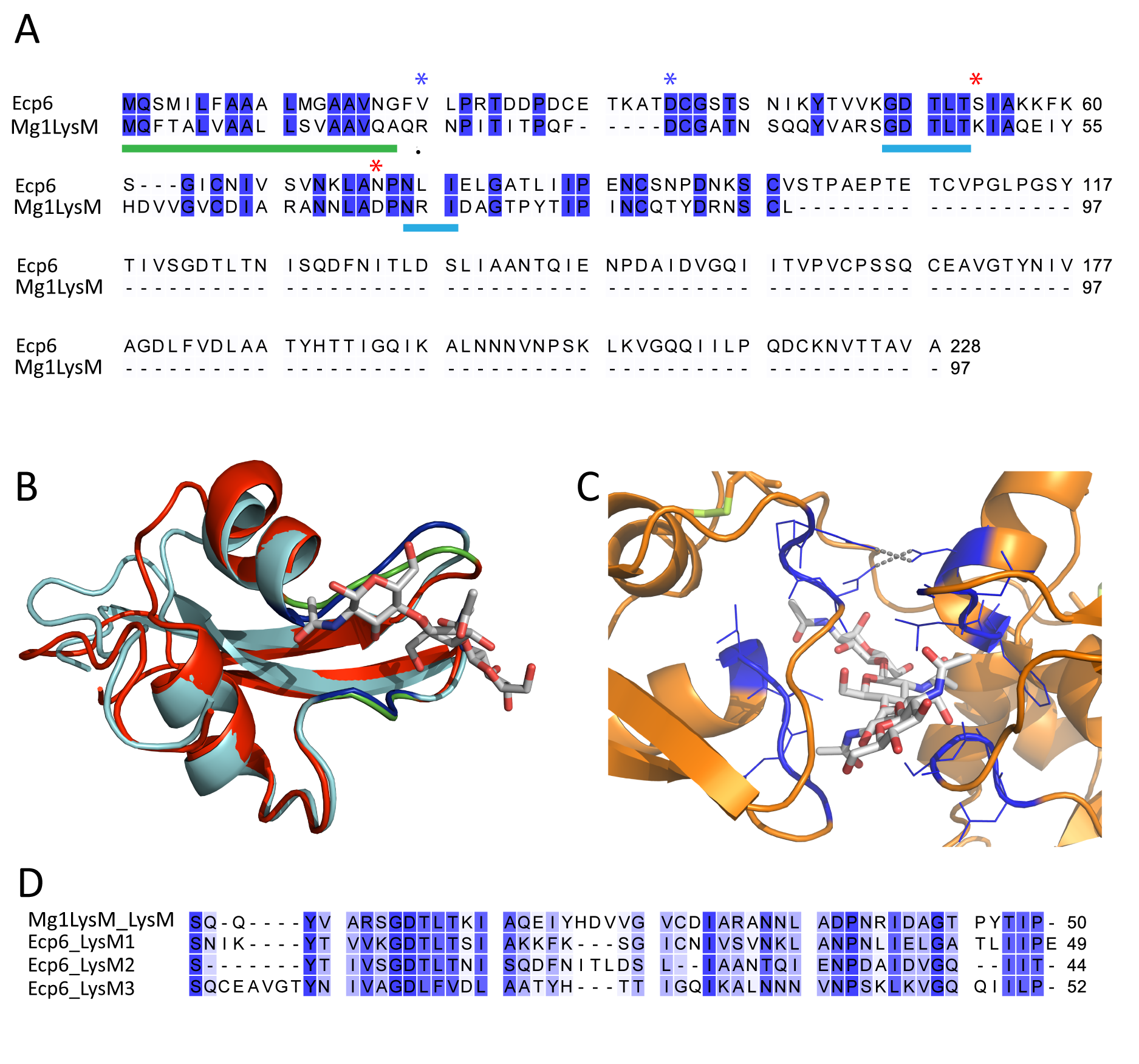

Supplement: S1 Fig — (A) Protein sequence alignment of Ecp6 and Mg1LysM. The two chitin binding loops of Mg1LysM are indicated with a blue line and the signal peptide with a green line. Red and blue asterisks indicate the position of the residues involved in the formation of salt bridges in the binding groove and in the dimerization surface, respectively. (B) Structural alignment of the LysM1 domain from Ecp6 (in blue) and the LysM domain from Mg1LysM (in red). The chitin trimer is shown in grey sticks. The chitin binding loops are shown in dark blue and green for LysM1 and for Mg1LysM, respectively. (C) Chitin binding pocket formed by LysM1 and LysM3 of Ecp6. In orange ribbons, a single molecule of Ecp6 is shown. The residues involved in chitin binding are shown as blue sticks. Hydrogen bonds between the two LysM domains are shown in grey. (D) Protein sequence alignment of the LysM domains of Ecp6 and Mg1LysM. (TIF) [file ppat.1008652.s001.tif]

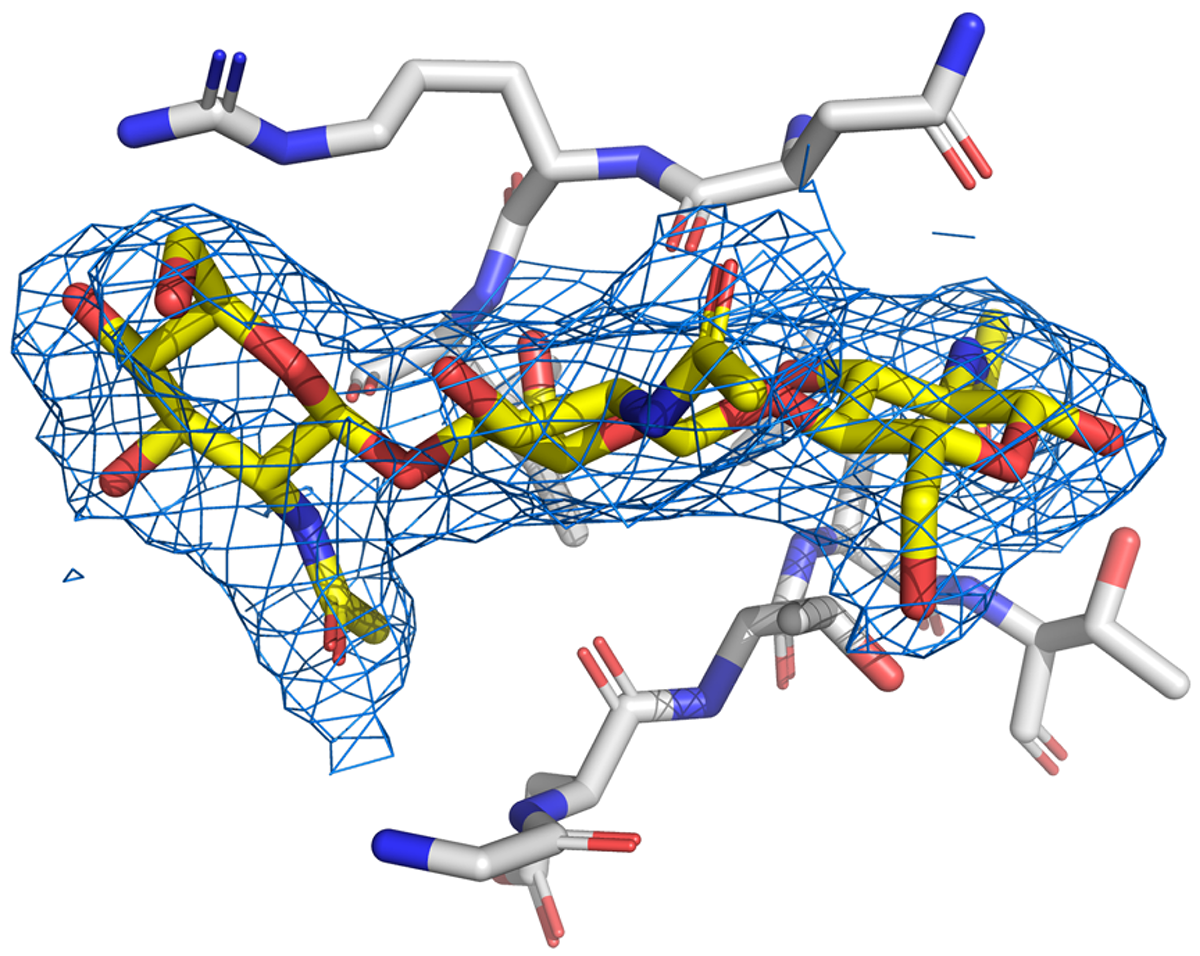

Supplement: S2 Fig — 2|F0|–|Fc| electron density map around the chitin trimer (carbon atoms coloured yellow) is contoured at 1 sigma above the mean. Amino acids of the chitin binding motif (26GDTLT30 and 56NRI58) are represented as sticks (carbon atoms coloured light-grey). (TIF) [file ppat.1008652.s002.tif]

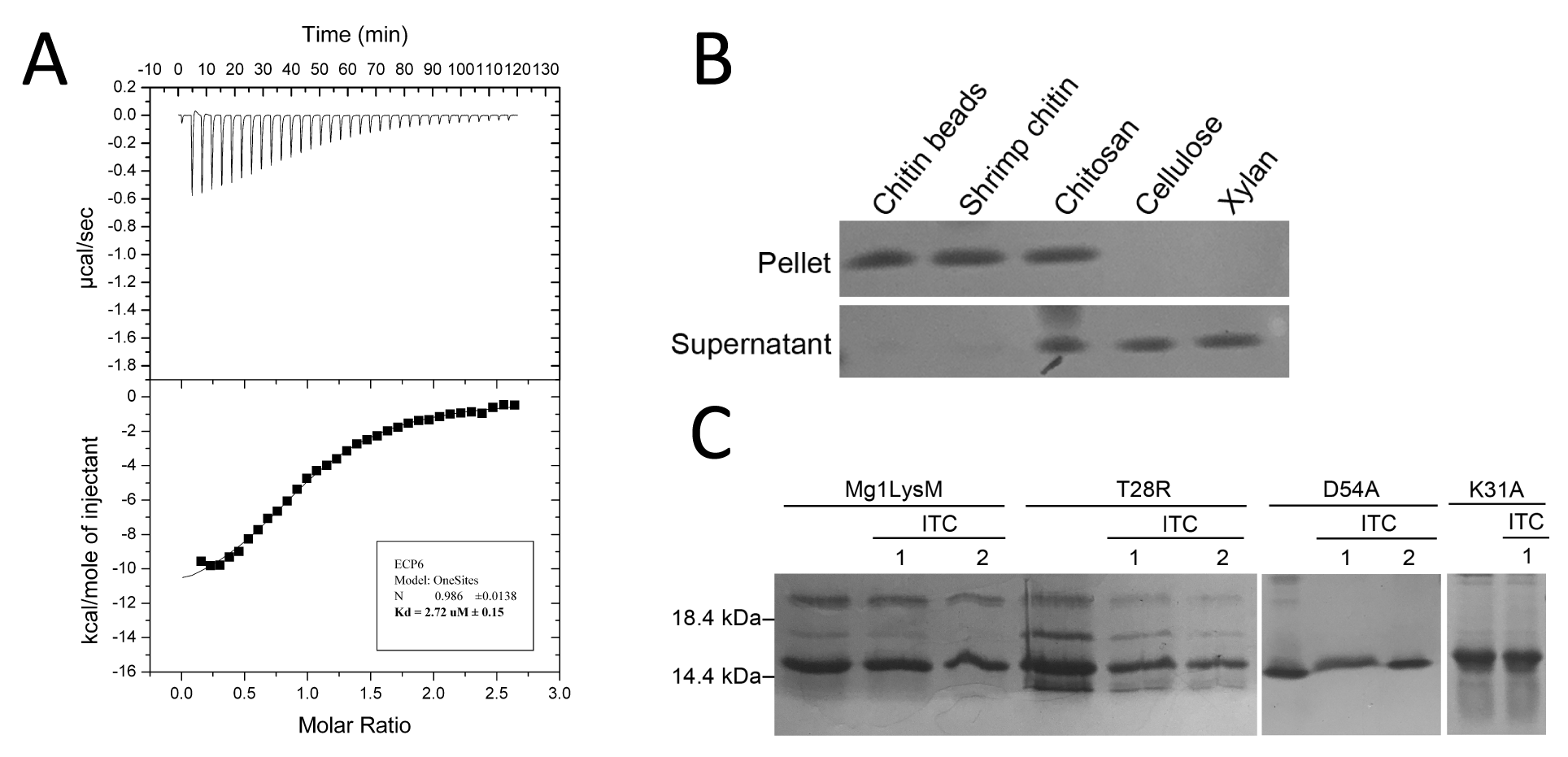

Supplement: S3 Fig — (A) Isothermal titration calorimetry of (GlcNAc)6 binding by Ecp6 produced in P. pastori. (B) Mg1LysM protein binds to insoluble chitin, but not to other carbohydrates. The purified Mg1LysM protein produced in E. coli was incubated with chitin beads, the insoluble carbohydrates shrimb chitin, chitosan, cellulose and xylan and centrifuged. Both the pellet and the supernatant were analyzed on protein gels. (C) Coomassie Brilliant Blue stained gel of Mg1LysM and mutant proteins before and after ITC assay. 1 and 2 indicate two independent ITC mesurements. (TIF) [file ppat.1008652.s003.tif]

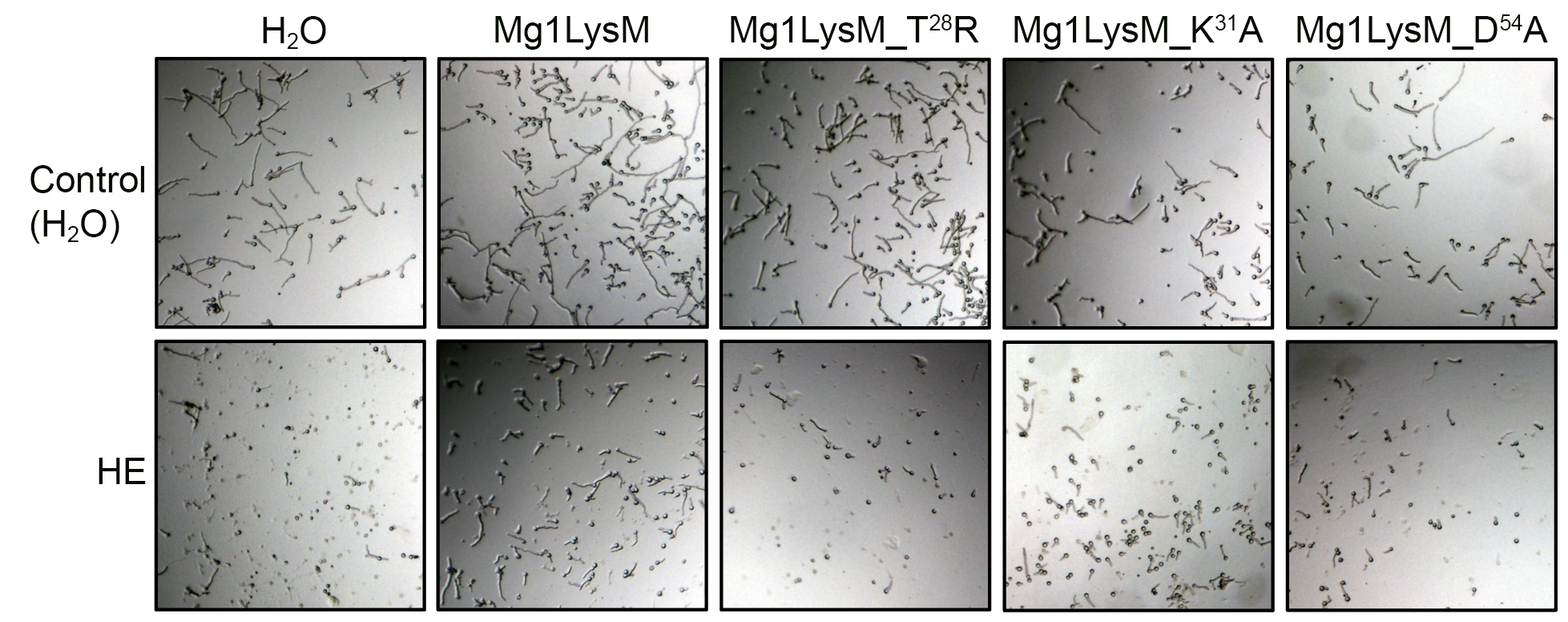

Supplement: S4 Fig — Microscopic pictures of Trichoderma viride grown in vitro in the absence or presence of wild-type or mutant Mg1LysM, 4 hours after addition of tomato hydrolytic enzymes (HE) that include chitinases, or water as control. (TIF) [file ppat.1008652.s004.tif]

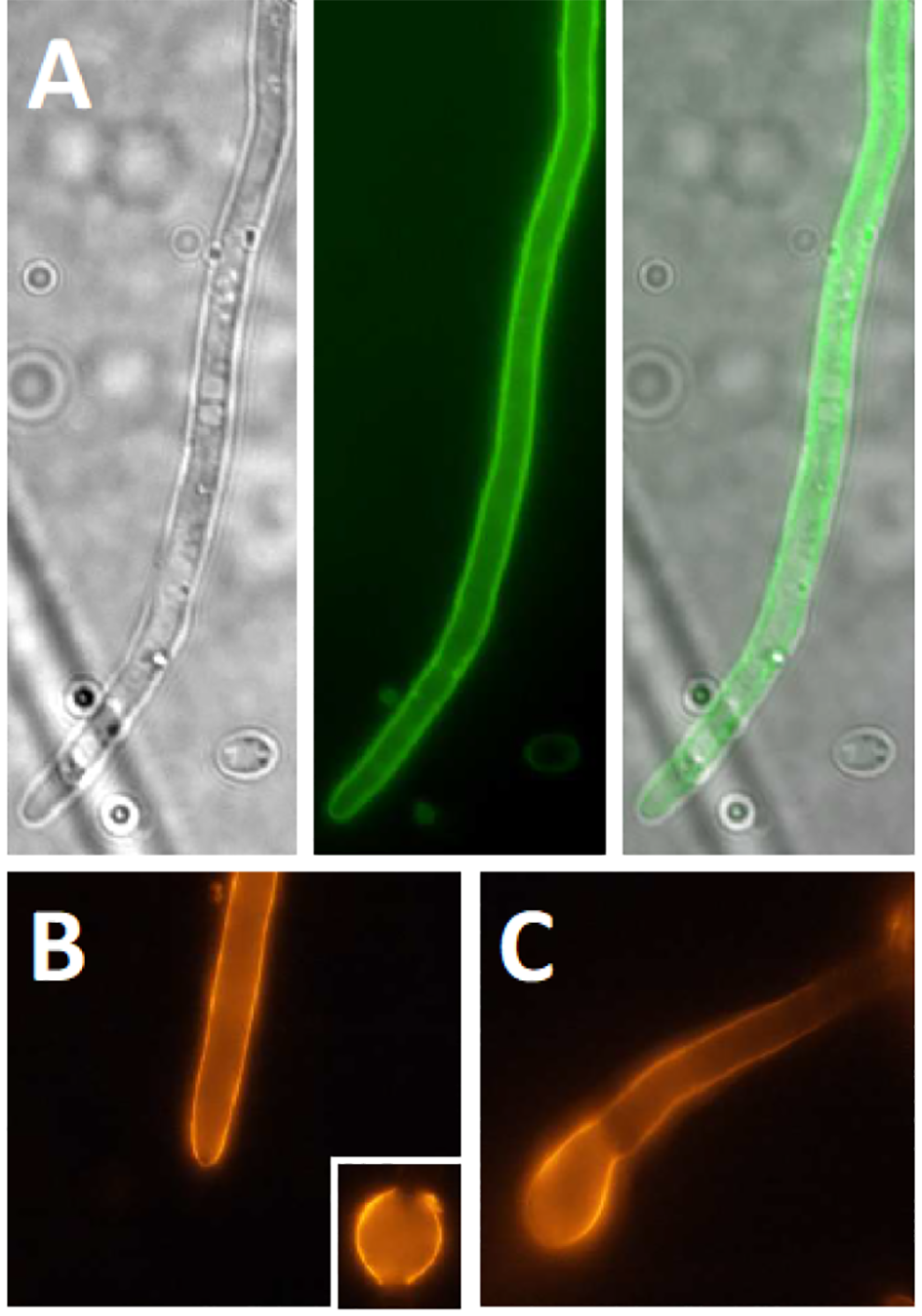

Supplement: S5 Fig — (A) Brightfield image (left), fluorescence image (middle) and the overlay image (right) of a hypha from an Ecp6-GFP transformant of Verticillium dahliae. The chitin-binding C. fulvum LysM effector Ecp6 (B) and chitin-binding effector Avr4 (C) that carries an invertebrate chitin-binding domain were labeled with the amine-reactive fluorescent dye BODIPY and incubated with Botrytis cinerea spores for 2–3 hours and observed with fluorescence microscopy. (TIF) [file ppat.1008652.s005.tif]
