# Supplementary material for: A secreted LysM effector protects fungal hyphae through chitin-dependent homodimer polymerization
Source: PLoS Pathog. 2020 Jun 23;16(6):e1008652. doi: 10.1371/journal.ppat.1008652 (PMC7337405; doi:10.1371/journal.ppat.1008652)
Supplement: S2 Table — (DOCX) [file ppat.1008652.s007.docx]

**S1 Table. Primers used in this study for cloning Mg1LysM and its mutants.**

| **Primer** | **Sequence** |
| --- | --- |
| Mg1LysM-pETSUMO-F | GCGCGCGAGCTCCAGCGGAATCCAATCACCATC |
| Mg1LysM-pETSUMO-R | GCGCGCAAGCTTctaTTAGAGGCAGCTGTTGCGGTCG |
| Mg1LysM-T28R-F | CGTCGCGCGCAGTGGAGACCGACTCACCAAGATCGCCC |
| Mg1LysM-T28R-R | CTTGGGCGATCTTGGTGAGTCGGTCTCCACTGCGCGCGACG |
| Mg1LysM-K31A-F | GGAGACACCCTCACCGCGATCGC |
| Mg1LysM-K31A-R | ATTTCTTGGGCGATCGCGGTGAG |
| Mg1LysM-D54A-F | CGAACAACCTGGCCGCCCCAAA |
| Mg1LysM-D54A-R | TCGATCCTGTTTGGGGCGGCCA |
