# Supplementary material for: A secreted LysM effector protects fungal hyphae through chitin-dependent homodimer polymerization
Source: PLoS Pathog. 2020 Jun 23;16(6):e1008652. doi: 10.1371/journal.ppat.1008652 (PMC7337405; doi:10.1371/journal.ppat.1008652)
Supplement: Attachment — Submitted filename: Response to reviewers.pdf [file ppat.1008652.s009.pdf]

Author responses to reviewer queries

Part II – Major Issues

Reviewer #1: 1. In figure 3, when examining Mg1LysM chitin-binding affinity by ITC assay, ECP6 should be included as a positive control.
Authors: We have now performed an additional ITC assay using Ecp6, basically repeating what we published before (Sanchez-Vallet et al., 2013, eLife) and we incorporated the results into the novel supplemental data Figure S3.

Reviewer #1: A second assay (e. g. in vitro pull-down) is also required to further confirm the Mg1LysM chitin-binding affinity.
Authors: We performed now an in vitro pull-down experiment as requested to confirm that Mg1LysM specifically binds chitin and not to other polysaccharides, such as cellulose. This information is also included in the novel supplemental data Figure S3 and highlights the capacity of Mg1LysM to bind long polymers of chitin, but not other polysaccharides as cellulose or xylan.

Reviewer #1: 2. In figure 6, it would be more convincible if the authors run the gel filtration chromatography to prove the chitin-induced Mg1LysM oligomerization and determine the molecular weight of the super-complex.
Authors: We have not performed gel filtration because of the amounts of protein that would be required for this assay. Furthermore, we are convinced that the super-complex is not of a particular size, as the complex can in principle extend infinitely. However, we do appreciate the point that an alternative strategy to demonstrate the oligomerization, besides the structure as observed in the crystal, and the DLS experiment, would be desirable. We have now resorted to centrifugation of Mg1LysM after incubation with short-chain chitin under the assumption that the protein will only be able to pellet if it polymerizes in a chitin-dependent manner. Indeed, we can now show that Mg1LysM can be pelleted after incubation with chitin, in contrast to Ecp6. These data are now presented as a novel Figure 7. With this, we believe to have proven polymerization to occur upon chitin binding in an independent manner.

Reviewer #1: 3. In Fig S4, the authors checked the subcellular location of ECP6 and Avr4, I think it is more important to examine the subcellular location of LysM1.
Authors: We have now also determined the subcellular localization of Mg1LysM after Bodipy labelling, demonstrating that Mg1LysM localizes to fungal cell walls. These data are now included as Figure 5A.

Reviewer #2: Figure 1. The authors showed a chitin-mediated dimer and chitin-independent dimer in the crystals. However, it is possible that the tetramers resulted from crystal packing. To rule out this possibility, gel-filtration or other biochemical assays of the Mg1LysM protein are needed to demonstrate formation of dimeric or tetrameric Mg1LysM in solution.
Authors: A close inspection of the contact points and of the structure in the crystal rules out that this structure is an artefact due to crystal packing. Ultimately, however, proof comes from the evidence for polymerization, as these polymers are built from the observed tetramers. We have not performed gel filtration because of the amounts of protein that would be required for this assay. Furthermore, we are convinced that the super-complex is not of a particular size, as the complex can in principle extend infinitely. However, we do appreciate the point that an alternative strategy to demonstrate the oligomerization, besides the structure as observed in the crystal, and the DLS experiment, would be

desirable. We have now resorted to centrifugation of Mg1LysM after incubation with short-chain chitin under the assumption that the protein will only be able to pellet if it polymerizes in a chitin-dependent manner. Indeed, we can now show that Mg1LysM can be pelleted after incubation with chitin, in contrast to Ecp6. These data are now presented as a novel Figure 7. With this, we believe to have proven polymerization to occur upon chitin binding in an independent manner. This, indirectly, proves that the observed tetramers in the crystal were genuine.

Reviewer #2: The crystal structure showed that the N-terminal tail is important for chitin-independent dimerization of Mg1LysM. Mutagenesis analyses are required to verify the importance of this region for dimerization.
Authors: We have tried to generate Mg1LysM mutants without the N-terminal tail by removing the first 12 aminoacids of the protein. However, we did not manage to purify the protein, as unsufficient amounts were produced. With the removal of shorter stretches of the tail we were not able to dusrupt the dimerization Alltogether, we believe that by disrupting the chitin-independent dimer, the protein becomes unstable, which is in agreement with the fact that the dimerization is required for the correct conformation of the protein. Furthermore, in the manuscript we now added the statement saying «Next, we attempted to evaluate the contribution of the ligand-independent Mg1LysM homodimerization to hyphal protection against chitinases. To this end, we pursued to produce an Mg1LysM mutant that lacked the 12-amino acid tail that is, besides the large protomer-protomer interface, responsible for ligand-independent homodimerization. Unfortunately, production of this mutant in the heterologous host P. pastoris was not successful as hardly any protein could be detected. The protein is apparently degraded either due to exposure of the hydrophobic residues (V40 and I68) located at the centre of the large (1113 Å2) homodimerization interface, or homodimerization is stringently required for proper folding of the protein.»

Reviewer #2: Figure 2. The structural diagram is unclear. The amino acids involved in the chitin recognition should be labeled. Also the mechanism of chitin binding should be compared with other LysM proteins to show unique or conserved binding patterns.
Authors: We have now modified Figure 2 as suggested and labelled the residues involved in chitin binding. In order to compare the mechanisms of chitin-binding with other LysM effectors, we showed the chitin-binding site of Ecp6 in S2 Fig. As previously demonstrated, in Ecp6 two LysM domains from the same monomer dimerize to bind chitin. In contrast to Mg1LysM, in Ecp6 there is no salt bridge between the LysM domains involved in chitin binding, which underpins that chitin-induced dimerization is stronger in Mg1LysM.

Reviewer #2: Figure 3. The stoichiometry between chitin and Mg1LysM was determined by the N value of ITC. The N values for WT (~0.5) and mutant proteins (~1.0) are different in the ITC assays. Monomeric K31A or D54A was proposed to bind to chitohexaose. Again biochemical assays are needed to demonstrate the monomeric state of these two mutant proteins. This is important, because data from such assays would correlate Mg1LysM polymerization with protection of hyphae against the hydrolytic activity of plant chitinases (Figure 5).
Authors: In our opinion it is obvious why the N value changes from 0.5 to 1 for the mutants: the ligand-induced dimerization no longer takes place. Furthermore, the use oft he mutants to draw conclusions on the correlation between polymerisation and hyphal protection is extremely complicated, as not only the dimerization may be affected, but also the chitin-binding ability because oft he altered affinity oft he mutants. This is a well-known downside oft he use of mutated proteins. Therefore, we can only conclude from

these experiments that chitin-binding of Mg1LysM is required to confer protection of cell walls against hydrolysis by plant enzymes. However, unfortunately, based on the mutants it is impossible to determine the contribution of the dimerization to the protection activity of Mg1LysM. This is also stated in the results section of the manuscript.

Reviewer #2: Figure 6. Data from DLS did agree with their oligomeric model of Mg1LysM. Additional methods should be used to verify this model. Ideally, negative staining of EM and ultracentrifugation analysis would provide more convincing evidence for the conclusion.
Authors: We have considered several strategies of trying to demonstrate polymerisation with an independent technology, taking two complications into account: the not unlimited amount of protein that we have at out disposal and the amount of chitin that would be required, but also the expectation that we will not obtain complexes of a particular size, as polymers can in principle extend infinitely. As explained above, we have attempted centrifugation of Mg1LysM after incubation with short-chain chitin with the expectation that we would be able to pellet protein polymers. If no polymers would be formed, such pellets cannot occur. We made use of short-chain chitin (a hexamer) to make sure that it is not the chitin that pellets and pulls the protein down, but also did the necessary controls to confirm. Indeed, we can now show that Mg1LysM can be pelleted after incubation with chitin, in contrast to Ecp6. These data are now presented as a novel Figure 7. With this, we believe to have proven polymerization to occur upon chitin binding in an independent manner.

Reviewer #3: 1. The claim of authors about D54A mutant no longer protected cell walls, suggesting that a ten-fold reduction of chitin-binding affinity is sufficient to disrupt the protective activity of Mg1LysM is skeptical. For example, why the disruption of a same pair of salt bridge (D54-K31) causes a much higher difference (338.9 μM vs 46.3 μM) in their chitin-binding affinities.
Authors: We have shown that the high binding affinity of Mg1LysM to chitin is critical for its biological activity. For this reason, a ten-fold reduction is likely to be enough to disrupt its function. By obtaining the mutants in the salt bridge, we aimed to investigate the relevance of chitin-mediated dimerization for protection against chitinases. Not only did the obtained mutants have a reduced binding affinity, they were also impaired in chitin-triggered dimerization (N value changes from 0.5 to 0.9). These two properties of Mg1LysM are critical for protecting fungal cell wall against the hydrolytic activity of chitinases. Since aspartic acid (D) and lysine (K) have different biochemical properties, we did not expect the same reduction in chitin-binding affinity when we mutagenized them to Alanine. After all, it is not only the salt bridge that we disrupt with the mutation, but also the amino acid in its context.

Reviewer #3: 2. It was hypothesized that LysM effectors that do not protect hyphae against chitinase hydrolysis would not display oligomerisation. The hypothesis was tested by DLS using Ecp6 in comparison to Mg1LysM. Analysis of additional LysM effectors is required to strengthen the hypothesis furthermore.
Authors: As we are limited in the amount of proteins we have at our disposal, and these assays require substantial protein amounts, we were not able to test more effectors of this type. Not all LysM effectors are readily produced heterologously. However, we have now tested another effector that is able to protect hyphae, RiSLM from the fungus Rhizophagus, and this effector again shows polymerisation in a chitin-dependent manner.

Reviewer #3: 3. This manuscript claimed that the disruption of ligand-independent homo-dimer

would result in loss of function for Mg1LysM. However, more direct evidence is needed to show such functional relevance.
Authors: As explained above, we pursued disruption of Mg1LysM dimerization by removing the N-terminal tail. Unfortunately, we failed in producing such mutants, most likely because the protein was unstable. We consider that obtaining mutants impaired in dimerization is a challenge due to the unwanted exposure of hydrophobic residues in the dimerization interface. Thus, the protein is apparently degraded either due to exposure of the hydrophobic residues (V40 and I68) located at the centre of the large (1113 Å2) homodimerization interface, or homodimerization is stringently required for proper folding of the protein. This is now explained in the results section.

Part III – Minor Issues: Editorial and Data Presentation Modifications

Reviewer #1: 1. To confirm the functional conservation of Mg1LysM1, the authors should check the chitin-binding affinity of several other Mg1LysM homologs from other isolates.
Authors: We have only been able to identify five non-synonymous mutations in the full length Mg1LysM protein. To test the impact of these polymorphisms on chitin binding, we heterologously produced two allelic variants of Mg1LysM that, collectively, carry the five non-synonymous mutations (see the newly added Fig 4C and S1 Table). Interestingly, a polysaccharide affinity precipitation assay revealed that, like the wild-type protein Mg1LysM, the two variants Mg1LysM_1E4 and Mg1LysM_3F4 still bind chitin (Fig 4D), suggesting that the allelic variants have retained their biological activity.

Reviewer #1: 2. In figure 1A, the recombinant Mg1LysM protein used for determination of the crystal structure was heterologously produced in the yeast Pichia pastoris. The authors should present a figure to show the protein expression of Mg1LysM.
Authors: The protein can be seen in the newly prepared supplementary Figure S3, more specifically in panel C.

Reviewer #1: 3. In line 160, there's no figure 1B and 1C. please check it.
Authors: We apologize for this error. This mistake has now been corrected in the text.

4. In figure 3, the recombinant protein used for the ITC assays should be shown by CBB staining. A second assay (e. g. in vitro pull-down) are required for confirm the Mg1LysM-chitin interaction. Ecp6 should be include as a positive control.
Authors: We have now included in supplementary Figure S3, an ITC experiment performed with Ecp6. In addition, we also performed an in vitro pull-down assay with Mg1LysM which shows that this protein binds to insoluble chitin, but not to other polysaccharides as cellulose. Finally, we included the CBB gel of the protein used for the ITC experiments.

Reviewer #2: (No Response)

Reviewer #3: (No Response)
