# Supplementary material for: A secreted LysM effector protects fungal hyphae through chitin-dependent homodimer polymerization
Source: PLoS Pathog. 2020 Jun 23;16(6):e1008652. doi: 10.1371/journal.ppat.1008652 (PMC7337405; doi:10.1371/journal.ppat.1008652)
Supplement: Attachment — Submitted filename: Response to reviewers-II.pdf [file ppat.1008652.s010.pdf]

Author responses to reviewer queries

Dear Editor,
Dear Hui-Shan,

Thank you very much for the time and consideration spent to evaluate out manuscript. I am happy to see that two of the referees are satisfied with the changes we have made to the manuscript, and also that most of the changes requested by the second referee, including those that were mentioned in a separate Word document are deemed satisfactory. However, the second referee still has two requests.

Part II – Major Issues
Reviewer #1: (No Response)
Reviewer #3: (No Response)
Reviewer #2: In the revision, the authors performed a polymerization assay in order to support chitin-induced oligomerzation of Mg1LysM. Unfortunately, the data from the assay are not sufficient to support the conclusion. They must present direct biochemical evidence for this using either gel filtration or EM negative staining. The former assay should be realistic, since the authors have obtained sufficient amount of protein for crystallization. Furthermore, gel filtration can easily tell whehter chitin binding induces dimerization of Mg1LysM.
Authors: We were asked in the reviews of our original submission to provide evidence for the proposed chitin-induced polymerisation in a second, independent manner besides the DLS experiments. Although to our opinion the DLS wás already a second manner, besides our observations in the crystal structure, we pursued another manner nevertheless. Thus, we demonstrated the validity of our observations by showing polymerisation in a centrifugation assay as an independent approach; an observation that cannot be explained otherwise than through chitin-mediated polymerization of Mg1LysM. First of all, I notice that none of the three referees questions this method, Furthermore, I also notice that none of the three referees, including referee 2, challenges the outcome or the conclusion we draw. Yet, without further explanation, referee 2 states "Unfortunately, the data from the assay are not sufficient to support the conclusion. They must present direct biochemical evidence for this using either gel filtration or EM negative staining. The former assay should be realistic…**". First of all, I do not see how DLS and the centrifugation approach are no direct biochemical evidence as the referee seems to suggest. And if these are not, how come that gel filtration is? Furthermore, there are three issues with gel filtration that this referee may have overlooked and that will obstruct his proposal: (1) the very large amounts of protein that are required and the very large amounts of chitin for saturation of the column that is required, (2) the fact that there will not be one uniform population of polymers, but a population of polymers of all kinds of sizes that will results in a smear rather than a peak, which will make that the analysis will be hard to perform and potential peaks hard to detect, and (3) the fact that large polymers will not migrate through the column but precipitate (as shown in the centrifugation assay), leading to clogging, and thus ruining, of the gel filtration column. Thus, the proposed method will simply not be possible. However, besides these practical concerns I would like to raise the question why we need yet another method to demonstrate what we have demonstrated before with multiple approaches, and what was deemed convincing by the two other referees who initially raised the same concern? Finally, it is worth mentioning that, besides through the additional centrifugation technique, we did demonstrate that our observations could be confirmed for another LysM effector, namely RiSLM1, in the revision of our manuscript, further solidifying our conclusions.**

Reviewer #2: It is unfortunate that deletion of the N-terminal tail made Mg1LysM unstable. But the authors should test if point mutations (R2, D12, I and T) can disrupt the interaction between the two N-terminal tails. Data from this or a similar experiment are also important to support their conclusion.
Authors: We have indeed shown in the manuscript that deletion of the N-terminal tail made Mg1LysM unstable. All observations suggest that the protein is not "happy" if the large hydrophobic plane that is normally covered by the dimerisation, is exposed so solvent. Our data really point towards the fact that mutants either still dimerize, and are thus useless, or no longer dimerize, and will be unstable. Making the mutants to come to this conclusion, again, will require a lot of time and effort to further support this notion. However, I would also like to encourage further thinking about the overall relevance of the question, because IF the protein would NOT occur as a dimer, it would NEVER be able to polymerize in the presence of chitin as it would only have a single chitin-binding site, and thus never be able to form a polymeric chain. Thus, our observation of chitin-induced Mg1LysM polymerization confirms that Mg1LysM undergoes chitin-independent dimerization. With that, the relevance of the request should be released.

Based on the arguments detailed above, we have not made any further changes to the manuscript. We sincerely hope that we have now convinced the referee and the editor that the remaining concerns have been sufficiently addressed.

Yours sincerely,
Bart Thomma
