# Supplementary material for: A secreted LysM effector protects fungal hyphae through chitin-dependent homodimer polymerization
Source: PLoS Pathog. 2020 Jun 23;16(6):e1008652. doi: 10.1371/journal.ppat.1008652 (PMC7337405; doi:10.1371/journal.ppat.1008652)
Supplement: Attachment — Submitted filename: Review comments.docx [file ppat.1008652.s008.docx]

The manuscript by Sanchez-Vallet et al. reported that the Mg1LysM effector protects fungal hyphae through chitin-dependent homodimer polymerization. Plant chitinase enzymes hydrolyze fungal cell wall to release chitin molecules, triggering immune responses, whereas plant pathogen effectors like Ecp6 competitively binds chitin oligomers with host immune receptors to suppress plant immune responses. Through determining crystal structure of Mg1LysM with chitin, the authors found that both chitin-dependent and -independent dimers exist in Mg1LysM tetramers. Thus, a superamolecular structure of chitin-induced oligomerization of chitin-independent Mg1LysM homodimers was proposed to explain how single LysM domain effector protects fungal cell walls against host chitinases. This is a very interesting model. In addition to the structural data presented, the authors should provide more evidence for the mechanism that Mg1LysM forms a contiguous oligomeric structure to protect fungal cell wall.

Specific comments:

Figure 1

The authors showed a chitin-mediated dimer and chitin-independent dimer in the crystals. However, it is possible that the tetramers resulted from crystal packing. To rule out this possibility, gel-filtration or other biochemical assays of the Mg1LysM protein are needed to demonstrate formation of dimeric or tetrameric Mg1LysM in solution. The crystal structure showed that the N-terminal tail is important for chitin-independent dimerization of Mg1LysM. Mutagenesis analyses are required to verify the importance of this region for dimerization.

Figure 2

The structural diagram is unclear. The amino acids involved in the chitin recognition should be labeled. Also the mechanism of chitin binding should be compared with other LysM proteins to show unique or conserved binding patterns.

Figure 3

The stoichiometry between chitin and Mg1LysM was determined by the N value of ITC. The N values for WT (~0.5) and mutant proteins (~1.0) are different in the ITC assays. Monomeric K31A or D54A was proposed to bind to chitohexaose. Again biochemical assays are needed to demonstrate the monomeric state of these two mutant proteins. This is important, because data from such assays would correlate Mg1LysM polymerization with protection of hyphae against the hydrolytic activity of plant chitinases (Figure 5).

Figure 6

Data from DLS did agree with their oligomeric model of Mg1LysM. Additional methods should be used to verify this model. Ideally, negative staining of EM and ultracentrifugation analysis would provide more convincing evidence for the conclusion.
