# Supplementary material for: A secreted LysM effector protects fungal hyphae through chitin-dependent homodimer polymerization
Source: PLoS Pathog. 2020 Jun 23;16(6):e1008652. doi: 10.1371/journal.ppat.1008652 (PMC7337405; doi:10.1371/journal.ppat.1008652)
Supplement: Attachment — Submitted filename: Comments.docx [file ppat.1008652.s011.docx]

*First of all, I do not see how DLS and the centrifugation approach are no direct biochemical evidence as the referee seems to suggest. And if these are not, how come that gel filtration is?"*

The two methods (DLS and centrifugation) can surely provide direct evidence for the conclusion, but not sufficient as originally commented. The reason for this is that chitin-dependent homodimer polymerization is the major conclusion of the manuscript as highlighted by the title. However, the Mg1LysM protein generated from *P. pastoris*. had a chitin oligomer (trimer in the crystal structure) bound but unlikely formed polymers with different sizes. The author can assay the *P. pastoris*-produced protein using DLS and ultracentrifugation.

*Furthermore, there are three issues with gel filtration that this referee may have overlooked and that will obstruct his proposal:*

*(1) the very large amounts of protein that are required and the very large amounts of chitin for saturation of the column that is required.*

The authors have assayed Mg1LysM interaction with chitohexaose using ITC, which actually requires more protein than gel filtration.

*(2) the fact that there will not be one uniform population of polymers, but a population of polymers of all kinds of sizes that will results in a smear rather than a peak, which will make that the analysis will be hard to perform and potential peaks hard to detect.*

As a matter of fact, if the authors can present the data as described using gel filtration, I will be convinced of the conclusion.

Additionally, if the authors assay the Mg1LysM protein purified from *P. pastoris* using gel filtration, will they expect similar profiles to those from the protein purified from bacteria together with chitohexaose?

*and (3) the fact that large polymers will not migrate through the column but precipitate (as shown in the centrifugation assay), leading to clogging, and thus ruining, of the gel filtration column.*

This could be a problem with gel filtration, but the pellets appeared to be formed slowly as overnight incubation was needed.

Did the protein also precipitate in the ITC assays?

*Thus, the proposed method will simply not be possible.*

This is not just doable but can also provide valuable information on the major conclusion.

*However, besides these practical concerns I would like to raise the question why we need yet another method to demonstrate what we have demonstrated before with multiple approaches, and what was deemed convincing by the two other referees who initially raised the same concern? Finally, it is worth mentioning that, besides through the additional centrifugation technique, we did demonstrate that our observations could be confirmed for another LysM effector, namely RiSLM1, in the revision of our manuscript, further solidifying our conclusions.*

To me, these have little to do with science. So I don’t feel obliged to respond to them. As a reviewer, I feel it a duty to provide the editor(s) with my comments and opinions on the manuscript. They may be inaccurate, but are surely not personal.

*Authors: We have indeed shown in the manuscript that deletion of the N-terminal tail made Mg1LysM unstable. All observations suggest that the protein is not "happy" if the large hydrophobic plane that is normally covered by the dimerisation, is exposed so solvent. Our data really point towards the fact that mutants either still dimerize, and are thus useless, or no longer dimerize, and will be unstable. Making the mutants to come to this conclusion, again, will require a lot of time and effort to further support this notion. However, I would also like to encourage further thinking about the overall relevance of the question, because IF the protein would NOT occur as a dimer, it would NEVER be able to polymerize in the presence of chitin as it would only have a single chitin-binding site, and thus never be able to form a polymeric chain. Thus, our observation of chitininduced Mg1LysM polymerization confirms that Mg1LysM undergoes chitinindependent dimerization. With that, the relevance of the request should be released.*

Many swapped dimers have been demonstrated by crystallography. Some are biologically relevant, while others are just artifacts resulting from crystal packing. As suggested by the authors, it might be possible that their dimer can’t tolerate any mutation in its dimerization interface. However, identification of loss-of-dimerization mutants would be very useful for verification of the model because such mutants are predicted to form a chitin-induced dimer but not an oligomer, if the proposed model is correct.
